# Marination of Beef With Unripe Grape (Koruk) Products at Different Holding Times: Quality Assessment

**DOI:** 10.1002/fsn3.70428

**Published:** 2025-06-13

**Authors:** Gulen Yildiz Turp, Ilkin Yucel Sengun, Gulden Kilic, Şeyma Nur Şeker, Berna Ozturk, Aysegul Kirmizigul Peker

**Affiliations:** ^1^ Ege University, Engineering Faculty Food Engineering Department Izmir Turkiye; ^2^ Alanya University, Art and Design Faculty, Gastronomy and Culinary Arts Department Alanya Antalya Türkiye; ^3^ Istanbul Nişantaşı University, Art and Design Faculty, Gastronomy and Culinary Arts Department İstanbul Türkiye

**Keywords:** grape, lipid oxidation, marinating, marination time, meat, microbial spoilage

## Abstract

The objective of this study was to examine the effects of utilizing unripe grape (koruk) juice (UGJ) and dried unripe grape pomace (DUGP) with thyme as marinade components and applying distinct marination holding times (2, 24, 48 h) on the physical, chemical, microbiological, and sensory properties of beef. pH, total acidity, salt content, water activity, and marinade absorption values of samples were affected by the usage unripe grape products (UGP) in marinade formulations and different marination holding times. UGP demonstrated efficacy in impeding oxidation at 48 h of marinating. The most efficacious treatment in reducing the counts of total mesophilic aerobic bacteria (TMAB) and total psychrotrophic aerobic bacteria (TPAB) in samples was achieved by marination with 50% UGJ for 48 h. The counts of *Pseudomonas* spp., Enterobacteriaceae, and LAB were below the detection limits for samples marinated with 50% UGJ and 50% UGJ with thyme and salt. If the marination process was conducted for 24 h, it was observed that all marinated sample groups exhibited significantly elevated scores for color, texture, and overall acceptance when compared to the control sample (*p <* 0.05). The overall acceptance scores of the samples marinated for 24 h with 25% and 50% unripe grape juice, as well as thyme and salt, were 6.6 and 6.7, respectively, on a 9‐point hedonic scale. It was thus concluded that UGJ and DUGP could be recommended as natural components in beef marination for the purpose of improving quality characteristics.

## Graphical abstract

The present study investigated the effects of unripe grape products with thyme and marination times (2, 24, 48 h) on beef properties. Marinating the samples for 48 h showed that grape products prevented oxidation and 50% unripe grape juice was the most effective treatment for reducing TMAB and TPAB counts. It was concluded that unripe grape products can be recommended as natural components in beef marinades to improve their quality.

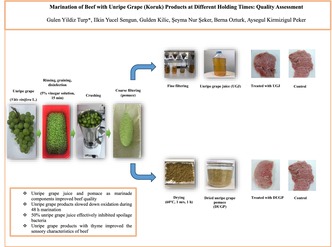

## 1 Introduction

Meat is subjected to a process known as marination, whereby it is immersed, injected, or tumbled for varying periods of time in aqueous solutions comprising salt, sugar, oils, herbs, and spices, phosphates, and organic acids. The objective of marination is to enhance the tenderness, flavor, juiciness, and yield of the meat product. Additionally, this process positively impacts the microbiological quality and shelf life of the meat.

Commercial marinades are frequently utilized in conjunction with acidic emulsions and a range of functional additives, including xanthan and guar gum, antimicrobial agents, flavor enhancers, pH‐lowering additives, and organic acids. The incorporation of acidic ingredients has been demonstrated to exert a pronounced influence on the tenderizing and flavor enhancement of marinated meat (Yusop et al. 2010; Augustyńska‐Prejsnar et al. 2023; Haraf et al. 2024; Wakita et al. 2024).

The effectiveness, quality, and safety of the meat are contingent upon the marination content and application parameters, including marination holding time (Ozturk and Sengun 2019; Sengun et al. 2021; Vişan et al. 2021; Unal et al. 2022). New processing and ingredient modification strategies based on the use of plant additives are currently being developed in meat technology with the objective of minimizing health concerns of consumers due to the potential adverse health effects of synthetic additives. Thus, the use of marinade ingredients derived from plants with low pH, antioxidant, and antimicrobial properties could prove an effective means of improving meat quality and meeting consumer expectations. For this reason, studies examining the effects of various plants on meat quality by using them instead of synthetic additives in the marination process have increased in recent years. These include citric acid, lemon juice, and porcupine juice in beef (Klinhom et al. 2011), pomegranate juice in chicken (Lytou et al. 2018), koruk products in beef (Ozturk and Sengun 2019) and poultry (Sengun et al. 2020), organic fruit vinegars in beef (Sengun et al. 2021), citric acid, lemon, and grapefruit juice in poultry (Unal et al. 2022), and black chokeberry, grape, and hawthorn vinegar‐based marination liquids in cow meat (Unal et al. 2023). The effects of the additives used in marination vary depending on the length of time the meat is kept in the marinade. In the limited number of studies examining the effect of the holding time of meat in marination solution on the properties of meat, it has been determined that meat quality changes significantly depending on the holding time (Kaewthong et al. 2021; Vişan et al. 2021; Karageorgou et al. 2023).

The unripe grape (koruk) juice, derived from unripe grapes ( *Vitis vinifera* L.), is utilized as an acidifying and flavoring agent in a variety of culinary applications, including salads, meals, and appetizers (Nikfardjam 2008; Alipour et al. 2012). The grape is a member of the *Vitaceae* family and is one of the most economically significant fruits cultivated globally, with an annual production of 68 million tons (Majeed et al. 2023). The thinning process is a common application in viticulture to improve the quality of table grapes (Gutierrez‐Gamboa et al. 2021; Wei et al. 2022). The purpose of thinning is to remove clusters that are unable to reach optimal ripeness, therefore encouraging the ripening of the remaining clusters on the plant. The thinned unripe grapes are left in the field to decay (Fia et al. 2022). Studies revealed that each year around 14436.16 kt of unripe grapes are discarded in vineyards around the world (Wei et al. 2022, 2023). The discarded unripe grapes lead to a considerable wastage of agricultural products and impose a significant environmental burden (Wei et al. 2021).

It has been demonstrated that unripe grape exhibits markedly higher levels of total phenolic and antioxidant activity in comparison to ripe grape (Doshi et al. 2006; Otağ and Kadakal 2015). During the ripening of grape, its weight continues to rise and the content of phenolic compounds declines since these compounds are mainly synthesized in the skin (Honisch et al. 2020). Thus, unripe grapes usually exhibit significantly higher levels of gallic acid, caffeic acid, catechin, and quercetin‐3‐O‐glucoside in comparison to ripened grapes (Nikfardjam 2008; Gutierrez‐Gamboa et al. 2021). Also, unripe grapes are rich in organic acids and other bioactive components (Wei et al. 2021). A number of studies have demonstrated the health benefits of unripe grape extracts, including improved serum cholesterol levels (Zibaeenezhad et al. 2012), reduced blood sugar (Gutierrez‐Gamboa et al. 2021) and the inhibition of cancer cell viability (Nasser et al. 2020; Wei et al. 2022).

One method of utilizing unripe grapes is the production of unripe grape juice. The unripe grape juice has been demonstrated to possess notable antioxidant and antimicrobial properties, largely attributed to its organic acid and phenolic compound content (Dupas de Matos et al. 2017; Turkmen et al. 2017). In addition, unripe grape juice has been demonstrated to effectively inhibit the growth of certain foodborne pathogens on a range of foods, including cucumber, parsley, beef, and poultry meat. This makes it a potential antimicrobial agent for use in food products (Karapinar and Sengun 2007; Ozturk and Sengun 2019; Sengun et al. 2019). The process of obtaining grape juice inevitably results in the generation of grape pomace, which is a waste product. The starting point of this study was to evaluate the possibility of using unripe grape juice, which is one of the evaluation areas of unripe grapes with notable antimicrobial and antioxidant properties, and the pomace produced as a waste in its production, in marinating beef to improve the quality of beef with natural additives. This study hypothesizes that unripe grape products enhance beef quality and reduce microbial spoilage and lipid oxidation. Therefore, the objective of this study was to examine the effects of marinades prepared with unripe grape juice, dried unripe grape pomace, and different marination holding times on the physical, chemical, microbiological, and sensory characteristics of beef.

## 2 Material and Methods

### 2.1 Preparation of Unripe Grape Juice and Dried Unripe Grape Pomace

Unripe grape samples ( *Vitis vinifera* L.), also known as koruk, were procured from Yesilyurt vineyards (Yediveren variety) in Izmir, Türkiye. Subsequently, the samples were rinsed briefly with tap water and grained. They were then soaked in a vinegar solution (5%, v/v) for 15 min to disinfect. Subsequently, the samples were crushed in a blender for 1 min at high speed (Waring Commercial Blender, New Hartford, Connecticut, USA). The crushed samples were filtered using cheesecloth and filter paper. The resulting liquid, designated as unripe grape juice (UGJ), was divided into portions and stored at −18°C until required for use. The resulting paste, comprising the fruit skins and seeds, was subjected to drying at 60°C and 1 m/s for 1 h in a tray dryer (TK Lab Model, Eksis Industrial Drying Systems, Türkiye) until reaching a moisture content of 3%. Thereafter, it was pulverized. The resulting powder, designated as “dried unripe grape pomace” (DUGP), was vacuum‐sealed in a Henkelman Boxer 42 vacuum packaging system (Holland) and stored at 4°C.

### 2.2 Experimental Design

The post‐rigor beef boneless rump roast was supplied by a local processor (Pinar Meat) and delivered under a cold chain to the Food Engineering Department at Ege University. The meat samples were cut transversally with a sterile knife into 100 g fillets with a thickness of 1 cm.

Marination application process of meat samples was carried out by using the static immersion method at 4°C. Meat samples were individually immersed in eight distinct marination liquids (ML) prepared as explained below and placed in separate covered glass containers. The ratio of meat to marinade was 1:2 (w/v). Two different marinade groups were produced by using unripe grape juice or dried grape pomace. Marinades containing unripe grape juice at 50% and 25% (v/v) or dried unripe grape pomace at 1% and 2% (w/v) were prepared with and without additives (salt, thyme) and water was used for dilution (Table 1). The beef samples that were treated solely with tap water (bc1) and those that were not treated at all (bc2) were designated as control samples. The samples were held in marinades at 4°C for 2, 24, and 48 h. These holding times were selected on the basis of the marinating times generally applied in marinating beef. The experiments were conducted on randomly selected samples.

**TABLE 1:** Beef sample groups with different marinade formulations.

Marination components	BML1	BML2	BML3	BML4	BML5	BML6	BML7	BML8	BC1	BC2
Unripe grape juice (mL)	50	50	25	25	—	—	—	—	—	—
Dried unripe grape pomace (g)	—	—	—	—	1	1	2	2	—	—
Water (mL)	50	50	75	75	100	100	100	100	100	—
Thyme (g)	—	0.1	—	0.1	—	0.1	—	0.1		
Salt (g)	—	1	—	1	—	1	—	1	—	—

### 2.3 Analysis of Marinated Beef Samples

The proximate composition, cooking loss, color properties, texture profile, and sensory properties of marinated meat samples were evaluated after cooking. The marinated meat samples were cooked for 10 min on each side in an electrical pan (Premier PPP 4045) with a minimum internal endpoint temperature of 75°C.

#### 2.3.1 Proximate Composition

The AOAC (2000) protocols were followed in measuring the moisture and ash contents of the marinated beef samples. Protein content was analyzed using the Kjeldahl method by using a conversion factor of 6.25 (Anonymous 1979). The Soxhlet extraction method was employed to assess the fat content by using petroleum ether in accordance with AOAC (2006).

#### 2.3.2 pH and Total Acidity

The measurement of pH was carried out on a 10 g uncooked marinated beef sample homogenized in 100 mL distilled water. The pH of the samples was measured using a pH meter (Inolab, WTW Series pH 720, Weilheim, Germany) (AOAC 2007). The total acidity of the uncooked marinated beef samples was determined by the titrimetric method (AOAC 2007).

#### 2.3.3 Salt Analysis

The salt content of the uncooked marinated beef samples was determined by the titrimetric method, as described by Kirk and Sawyer (1991).

#### 2.3.4 Water Activity

The water activity (a_w_) values of the uncooked marinated beef samples were determined using a water activity device (Testo 400, Germany).

#### 2.3.5 Marinade Absorption

Prior to and following the marinating process, beef sample weights were recorded. The marinade absorption of the marinated beef samples was calculated using Equation (1).

(1) $$\text{Marinade absorption}\;\left(\%\right)=\left[\left(A_{w}-B_{w}\right)/B_{w}\right]\times 100$$

where A_w_ is the weight after marination, and B_w_ is the weight before marination.

#### 2.3.6 Cooking Loss

Changes in weight of samples before and after cooking were used to calculate cooking loss (Rodrigues et al. 2016). The cooked marinated beef samples were cooled to room temperature for 30 min and were reweighed to calculate the cooking loss Equation (2).

(2) $$\text{Cooking loss}\;\left(\%\right)=\left[\left(B_{w}-A_{w}\right)/B_{w}\right]\times 100$$

where A_w_ is the weight after cooking, B_w_ is the weight before cooking.

#### 2.3.7 Thiobarbituric Acid Reactive Substances Analysis

The lipid oxidation of marinated uncooked beef samples was determined in accordance with the methodology delineated by Witte et al. (1970). The absorbances of the samples were quantified spectrophotometrically (Agilent Technologies, Carry 60 UV–Visible, United Kingdom) at 532 nm. The results were expressed as 2‐thiobarbituric acid reactive substances (TBARS) in mg of malonaldehyde (MDA) per kg of sample.

#### 2.3.8 Color Properties

The HunterLab Colorflex (CFLX 45–2 Model Colorimeter, HunterLab, Reston, VA, Port Diameter/View Diameter: 31.8 mm, illuminated/25.4 mm measured; directional annular 45° illumination/0° viewing; light source: pulsed xenon lamp) was employed to ascertain the chromatic characteristics of the marinated cooked beef samples. To calibrate the equipment, a black glass and a white tile were utilized. The color coordinates in CIE L*, a*, and b* were recorded on the external surfaces of the samples. L* represents brightness, a* represents green/red, and b* represents blue/yellowness (Kramer and Twigg 1984). Color measurements were performed on the outside surfaces of the cooked samples. Four readings were obtained for each sample, and three samples were assessed for each sample group.

#### 2.3.9 Texture Profile Analysis

The effects of marinades prepared with unripe grape products and different marination holding times on textural properties of cooked marinated beef samples were determined. A texture analyzer (TA‐XT2, Stable Micro Systems, Scarsdale, NY) was employed to conduct texture profile analysis (TPA) via the twofold compression approach. A cylindrical plate (25 cm in diameter) and power cell (5 kg) were utilized. The cooked marinated beef samples were shaped into a cubic form with dimensions of 1 × 1 × 1 cm^3^. The sample was compressed twice at a rate of 5 mm/s, a distance of 5 mm, and a 5‐s delay between the descents. The textural attributes of cohesiveness, springiness, hardness, gumminess, resilience, and chewiness were all measured. Texture profile parameters were calculated from the force vs. deformation curves obtained. Total compression area was also calculated as the total work required for the double compression of the sample (Icier et al. 2014). At least 10 measurements were performed for each treatment.

#### 2.3.10 Microbiological Analysis

A 25 g sample of marinated beef was combined with peptone water (225 mL of 0.1%, w/v) (PW, pH 6.8 ± 0.2, Oxoid‐CM0009) in a sterile filter bag (BioMérieux, Ref. 80,015) and homogenized in a Stomacher (Stomacher Lab‐Blender 400, Seward Medical, UK) for 1 min. The samples were prepared using serial 10‐fold dilutions of PW.

The counts of TMAB, TPAB, and Enterobacteriaceae were determined using the TEMPO system (BioMerieux, France). Following the appropriate serial dilution, the samples (0.1 mL) were separately transferred into TEMPO Aerobic Count (AC) (BioMerieux, Ref. 411,113) media that had been reconstituted with sterile distilled water (3.9 mL). The filled TEMPO AC cards (BioMerieux, Ref. 411,113) were used to determine the TMAB and TPAB counts. The samples were incubated at 35°C ± 2°C for 48 h for TMAB counts and at 7°C for 12 days for TPAB counts (FDA‐BAM (Food and Drug Administration‐Bacteriological Analytical Manual) 2001; Gilliand et al. 1976). Similarly, the samples (0.1 mL) were transferred into TEMPO Enterobacteriaceae (EB) (BioMerieux, Ref. 80,003) medium, which was reconstituted with sterile distilled water (3.9 mL). The filled TEMPO EB cards (BioMerieux, Ref. 80,003) were employed for the determination of Enterobacteriaceae counts following a 22–27 h incubation period at 35°C, in accordance with the ISO 21528‐2:2017 standard.

For the enumeration of *Pseudomonas* spp., an aliquot (0.1 mL) was spread plated on Glutamate Starch Phenol Red (GSP) Agar (pH 7.1–7.3, Merck‐M110230) and incubated at 30°C for 48 h (Emiroglu et al. 2010). The enumeration of lactic acid bacteria (LAB) was conducted on Man Rogosa and Sharp (MRS) Agar (pH 6.2 ± 0.2, Merck‐VM040261) using the double‐layer technique. The incubation was performed at 30°C for 3–5 days, in accordance with the ISO 15214:1998 standard.

#### 2.3.11 Sensory Evaluation

Marinated cooked beef samples were evaluated by the attendance of 10 panelists consisting of staff members and students, aged between 20 and 35, from the Ege University Food Engineering Department. The panel was conducted in individual booths at the sensory evaluation laboratory of Ege University in accordance with ISO (International Organization for Standardization) (2007) standards. A nine‐point hedonic scale (9 = extremely like, 1 = extremely dislike) was employed to assess the appearance, color, texture, flavor, and overall acceptance characteristics of the beef samples (Altug Onogur and Elmaci 2015). A random three‐digit code was assigned to each sample. The samples were provided to the panel immediately after the cooking process, accompanied by bread and room temperature water.

### 2.4 Statistical Analysis

The effects of different formulations prepared with UGJ, DUGP, and marination holding time periods (2, 24, and 48 h) on the physical, chemical, microbiological, and sensory properties of meat samples were examined using one‐way ANOVA. The differences between the means were examined using Duncan's Multiple Range test. For each evaluation, a significance level of *p* < 0.05 was employed. The data were analyzed using SPSS software version 20 SPSS (2011). The entire trial was replicated three times at various times. The results of the analysis were presented in tables and figures as mean values and standard error.

## 3 Results and Discussion

### 3.1 Effect of Marination on Proximate Composition

The effects of unripe grape products and marination time on the proximate composition of the cooked marinated meat samples are presented in Table 2.

**TABLE 2:** Influence of marinade formulations and marination holding times on proximate composition of beef samples.

Time		Samples									
		BML1	BML2	BML3	BML4	BML5	BML6	BML7	BML8	BC1	BC2
Moisture (%)	2 h	65.21 ± 1.29^bc^	65.16 ± 0.85^bc^	62.40 ± 0.16^a,A^	64.32 ± 0.66^abc,B^	64.76 ± 0,90^abc^	64.28 ± 0.79^abc^	63.35 ± 1.05^ab^	66.41 ± 0.67^c^	62.96 ± 0.43 ^ab,B^	64.62 ± 0.74^abc^
	24 h	67.02 ± 1.24^d^	62.58 ± 1.66^abc^	63.51 ± 0.79^bc,A^	60.84 ± 0.92^ab,A^	63.72 ± 1.07^bc^	65.29 ± 1.02^cd^	63.96 ± 0.94^bcd^	65.24 ± 1.09^cd^	60. 12 ± 0.54^a,A^	63.56 ± 0.70^bc^
	48 h	68.23 ± 2.09^d^	65.75 ± 1.38^cd^	65.13 ± 0.42^bcd,B^	60.99 ± 0.89 ^a,A^	63.67 ± 0.94^abc^	63.38 ± 0.64^abc^	62.32 ± 1.20^ab^	63.62 ± 0.57^abc^	62.42 ± 0.72^abc,B^	63.05 ± 0.25^abc^
Fat (%)	2 h	2.08 ± 0.09^a^	3.54 ± 0.55^bc^	2.70 ± 0.27^abc^	2.74 ± 0.33^abc,AB^	3.34 ± 0.23^bc^	3.40 ± 0.27^bc^	3. 02 ± 0.28^abc,B^	2.65 ± 0.37^abc^	2.44 ± 0.26^ab^	3.60 ± 0.48^c^
	24 h	2.59 ± 0.28	2.58 ± 0.52	3.47 ± 0.42	3.37 ± 0.43^B^	2.30 ± 0.58	3.09 ± 0.39	1.90 ± 0.22^A^	2.01 ± 0.27	2.94 ± 0.51	2.19 ± 0.44
	48 h	2.93 ± 0.44^ab^	4.30 ± 0.38^c^	3.68 ± 0.40^bc^	2. 01 ± 0.12^a,A^	2.86 ± 0.17^ab^	3.58 ± 0.40^bc^	2.16 ± 0.16^a,A^	2.95 ± 0.39^ab^	3.08 ± 0.55^ab^	2.83 ± 0.19^ab^
Protein (%)	2 h	34.72 ± 0.54^bcd^	33.09 ± 0.74^ab^	33.64 ± 0.55^ab^	33.16 ± 0.40^ab,A^	32.40 ± 1.08^a^	35.09 ± 0.47^bcd^	34.30 ± 0.61^abc,A^	32.47 ± 1.21^a^	36.17 ± 0.29^cd^	36.74 ± 0.55^d^
	24 h	31.30 ± 1.06^a^	34.28 ± 1.52^bc^	34.15 ± 1.22^bc^	36. 64 ± 0. 30^cd,B^	34.72 ± 0.46^bcd^	35.37 ± 0.26^bcd^	35.17 ± 0.21^bcd,AB^	31.83 ± 0.78^ab^	33.45 ± 0.70^cd^	36.59 ± 0.40^d^
	48 h	31.85 ± 1.67^a^	33.25 ± 1.77^abc^	33.39 ± 0.95^abcd^	35.60 ± 0.87^bcd,B^	32.81 ± 0.81^ab^	35.33 ± 0.43^bcd^	36.27 ± 0.37 ^cd,B^	33.27 ± 1.01^abcd^	36.59 ± 0.37^d^	35.76 ± 0.49^bcd^
Ash (%)	2 h	1.28 ± 0. 02^ef,C^	1.15 ± 0.04^bcde^	1.20 ± 0.02^cdef,C^	1.31 ± 0.03^f,C^	0.85 ± 0.09^a^	1.15 ± 0.02^bcde,B^	1.02 ± 0.05^b,C^	1.07 ± 0.04^bc,B^	1.23 ± 0.02^def,C^	1.12 ± 0.06^bcd^
	24 h	0.79 ± 0.03^a,B^	1.52 ± 0.40^b^	0.85 ± 0.04^a,B^	1.09 ± 0.03^a,B^	0.67 ± 0.06^a^	0.80 ± 0.06^a,A^	0.78 ± 0.05^a,B^	0.83 ± 0.06^a,A^	0.71 ± 0.02^a,B^	0.91 ± 0.11^a^
	48 h	0.62 ± 0.09^a,A^	0.92 ± 0.05^b^	0.52 ± 0.08^a,A^	0.89 ± 0.04 ^b,A^	0.66 ± 0.15^a^	0.68 ± 0.19^a,A^	0.54 ± 0.12^a,A^	0.66 ± 0.22^a,A^	0.58 ± 0. 08^a,A^	0.88 ± 0.29^b^

*Note:* a–f, mean values with different lower case differ significantly among samples (*p* < 0.05). A–C, mean values with different upper case differ significantly among marination times (*p* < 0.05).

The marination of meat in solutions of higher acid and salt concentrations has been shown to result in a greater moisture uptake (Aktas and Kaya 2001). The BML1 sample, which contained a high level of UGJ, exhibited a higher moisture content than the control samples at the end of the 24 and 48 h marination periods. All of the samples marinated with DUGP and UGJ without salt and thyme (BML1, BML3, BML5, BML6, BML7, BML8) exhibited higher moisture contents than the control sample bc1 at the conclusion of the 24 h marination period (*p* < 0.05). In a similar study, marination of pork with different concentrations of yellow mombin ( *Spondias mombin* L.) for 20 h resulted in significantly higher moisture contents in samples compared to the control sample (Beltrán‐Cotta et al. 2023). In line with these results, the moisture content of pork loin samples marinated with different ratios of grape pomace powder was detected as significantly higher than that of the control sample (Lee et al. 2017).

The effect of different marination times on moisture contents of the marinated samples was observed to be insignificant (*p* > 0.05), with the exception of BML3 and BML4. It was determined that the moisture content of the BML3 sample increased significantly when the marination time increased from 24 h to 48 h, while the moisture content of the BML4 sample decreased significantly after 2 h. The presence of thyme and salt in the BML4 formulation, which differed from that of BML3, was thought to influence the decrease in moisture content with increasing marination time. An increase in marination time from 24 to 48 h may cause structural changes in meat proteins due to the combined effects of salt and the acidic properties of UGJ, resulting in a decrease in water retention of the meat structure. Therefore, it is thought that the amount of water retained by the BML4 sample decreases when the marination time exceeds 24 h. The synergistic effect of the marinating and aging process has been shown to result in the structural weakening of diverse proteins, with distinct functional properties (Mirhaj et al. 2022; Javan et al. 2025).

The fat content of BML2 was markedly elevated in comparison to all other samples, with the exception of BML3 and BML6, at the conclusion of the 48 h (*p* < 0.05). The impact of diverse marination additives on the proximate composition of beef may fluctuate contingent on the intrinsic characteristics of these additives. In a study, the influence of marination in three distinct dealcoholized red wines over a 48 h on the proximate composition of beef was found to be inconsequential (Arcanjo et al. 2019).

The marination time had no effect on the fat and protein content of the samples, with the exception of BML4 and BML7. The ash content was highest in the BML2 and BML4 samples, which were marinated for 48 h with thyme and salt in addition to UGJ. This indicates that these samples had a higher mineral content than the others. Similar to this result, Ortega‐Heras et al. (2020) detected the highest ash value in the sample with the highest grape pomace seasoning (2%) and salt (2%) compared to other samples which include lower levels of these additives.

### 3.2 Effect of Marination on pH, Total Acidity, Salt Content and Water Activity

The pH of marinated meat is significantly affected by the type and ratio of additives in the marinade solution. The pH of the meat samples ranged between 4.05 (BML1) and 5.41 (bc1).

The lowest pH was observed in meat samples marinated with UGJ, which differed significantly from the samples with DUGP and the control (*p* < 0.05) (Table 3). In line with this result, the highest acidity values were detected in BML1 samples containing the highest amount of UGJ during all of the marination times (*p <* 0.05).

**TABLE 3:** Influence of marinade formulations and marination holding times on pH values, total acidity, salt content, and water activity of beef samples.

Time		Samples									
		BML1	BML2	BML3	BML4	BML5	BML6	BML7	BML8	BC1	BC2
pH	2 h	4.17 ± 0.01^a,A^	4.05 ± 0.00^b,A^	4.29 ± 0.01^c,A^	4.26 ± 0.00^d,A^	4.67 ± 0.02^e,A^	4.91 ± 0.01^f,A^	4.78 ± 0.01^g,A^	4.89 ± 0.00^f,A^	5.18 ± 0.01^h,A^	5.23 ± 0.01^ı,A^
	24 h	4.35 ± 0.01^a,B^	4.33 ± 0.00^b,B^	4.49 ± 0.00^c,B^	4.51 ± 0.00^c,B^	4.94 ± 0.01^d,B^	5.16 ± 0.00^e,B^	5.01 ± 0.01^f,B^	5.03 ± 0.01^g,B^	5.21 ± 0.01^h,B^	5.25 ± 0.01^ı,A^
	48 h	4.09 ± 0.01^a,C^	4.10 ± 0.00^a,C^	4.30 ± 0.01^b,A^	4.47 ± 0.00^c,C^	4.97 ± 0.00^d,B^	5.36 ± 0.01^e,C^	5.14 ± 0.01^f,C^	5.19 ± 0.01^g,C^	5.41 ± 0.01^h,C^	5.28 ± 0.01^ı,B^
Total acidity (g LA/100 mL)	2 h	0.29 ± 0.00^a,A^	0.21 ± 0.01^b,A^	0.12 ± 0.00^c,A^	0.10 ± 0.01^cd,A^	0.06 ± 0.01^e,A^	0.06 ± 0.00^e,A^	0.09 ± 0.01^d,A^	0.10 ± 0.00^cd,A^	0.10 ± 0.01^cd,A^	0.10 ± 0.00^cd,A^
	24 h	0.20 ± 0.00^a,B^	0.15 ± 0.01^b,B^	0.09 ± 0.01^c,B^	0.09 ± 0.00^c,A^	0.06 ± 0.00^de,A^	0.05 ± 0.00^ef,A^	0.04 ± 0.00^f,B^	0.06 ± 0.00^de,B^	0.05 ± 0.00^ef,B^	0.07 ± 0.00^d,B^
	48 h	0.17 ± 0.01^a,C^	0.13 ± 0.00^b,C^	0.07 ± 0.01^c,C^	0.06 ± 0.00^ce,B^	0.03 ± 0.00^d,B^	0.05 ± 0.00^ef,A^	0.07 ± 0.01^c,C^	0.06 ± 0.00^ce,B^	0.04 ± 0.00^df,B^	0.09 ± 0.01^g,C^
Salt (%)	2 h	0.40 ± 0.02^c,A^	0.77 ± 0.04^g^	0.33 ± 0.03^ab^	0.59 ± 0.02^e,A^	0.47 ± 0.01^d,A^	0.69 ± 0.01^f,A^	0.43 ± 0.01^cd,A^	0.73 ± 0.03^fg,A^	0.30 ± 0.01^a,A^	0.39 ± 0.02^bc,A^
	24 h	0.44 ± 0.07^c,A^	0.83 ± 0.04^e^	0.30 ± 0.03^b^	0.61 ± 0.07^d,A^	0.49 ± 0.00^c,AB^	0.90 ± 0.01^e,B^	0.45 ± 0.01^c,AB^	0.81 ± 0.02^e,B^	0.20 ± 0.03^a,A^	0.43 ± 0.03^c,A^
	48 h	0.62 ± 0.07^c,B^	0.91 ± 0.01^de^	0.29 ± 0.04^a^	0.82 ± 0.05^d,B^	0.51 ± 0.01^bc,B^	1.04 ± 0.00^e,C^	0.48 ± 0.01^b,B^	0.93 ± 0.01^de,C^	0.18 ± 0.01^a,B^	0.56 ± 0.03^bc,B^
Water activity	2 h	0.99 ± 0.00^c^	0.99 ± 0.00^c^	0.99 ± 0.00^c^	0.99 ± 0.00^c^	0.95 ± 0.01^a^	0.95 ± 0.00^a^	0.97 ± 0.01^b^	0.96 ± 0.00^b^	0.99 ± 0.00^c^	0.98 ± 0.00^c^
	24 h	0.99 ± 0.00^c^	0.99 ± 0.00^c^	0.98 ± 0.00^c^	0.98 ± 0.00^c^	0.94 ± 0.01^a^	0.95 ± 0.01^a^	0.96 ± 0.01^b^	0.96 ± 0.00^b^	0.99 ± 0.00^c^	0.98 ± 0.00^c^
	48 h	0.99 ± 0.00^c^	0.99 ± 0.00^c^	0.99 ± 0.00^c^	0.99 ± 0.00^c^	0.96 ± 0.01^a^	0.95 ± 0.01^a^	0.96 ± 0.01^a^	0.97 ± 0.00^b^	0.99 ± 0.00^c^	0.98 ± 0.00^bc^

*Note:* a–ı, mean values with different lower case differ significantly among samples (*p* < 0.05). A–C, mean values with different upper case differ significantly among marination times (*p* < 0.05).

Abbreviation: LA, lactic acid.

During the marination period, the pH of the samples underwent a change contingent upon the unripe grape product under consideration. The application of DUGP resulted in a notable increase in pH levels over the course of 48 h, whereas UGJ demonstrated a distinct rise at the 24 h mark, followed by a notable decline at the 48 h mark potentially due to the buffering effect (*p* < 0.05). Grape juice has a strong buffering capacity (Touyz and Nassani 2018). In a study conducted by Kargiotou et al. (2011), the addition of red wine‐based marinades and soy sauce to raw beef resulted in a significant reduction in pH compared to the control sample. Similarly, in another study, the pH of meat samples marinated in red wine and wine containing 0.3% thyme essential oil was found to be decreased in the range of 0.92–1.27 and 0.89–1.30 units, respectively, in comparison to non‐marinated samples (Nisiotou et al. 2013).

The salt content of all marinated samples exhibited a notable increase during the marination process, with the exception of BML2 and BML3 samples, which demonstrated a statistically significant difference (*p* < 0.05). The utilization of DUGP resulted in a notable reduction in the water activity values of the samples (*p* < 0.05). Previous research has demonstrated that the MLs prepared with acidic solutions have an impact on the pH, total acidity, and water activity values of the meat samples (Kargiotou et al. 2011; Nisiotou et al. 2013; Ozturk and Sengun 2019).

### 3.3 Effect of Marination on Marinade Absorption, Cooking Loss and TBARS Values

The capacity of meat products to absorb marinades to a considerable extent is a sought‐after quality, both in terms of the evolution of product characteristics and the optimization of processes. The marinade absorption values of the samples marinated with DUGP were found to be significantly higher than those of the other samples, with the exception of BML6, at the end of the 48 h marination period (*p* < 0.05) (Table 4). At both 24 and 48 h of marination, BML1 exhibited the highest marinade absorption values relative to the other samples. Beef samples should be marinated with UGJ for a minimum of 24 h, with 48 h being the optimal marination time for achieving the highest marinade absorption.

**TABLE 4:** Influence of marinade formulations and marination holding times on marinade absorption, cooking loss, and TBARS values of beef samples.

Time		Samples									
		BML1	BML2	BML3	BML4	BML5	BML6	BML7	BML8	BC1	BC2
Marinade absorption (%)	2 h	0.03 ± 0.62^bc,A^	−0.11 ± 0.29^bc,A^	−1.11 ± 0.30^abc,A^	−3.30 ± 1.78^a,A^	−2.45 ± 0.57^ab^	0.09 ± 0.41^bc^	−1.67 ± 0.87^abc^	−0.56 ± 0.45^abc^	1.09 ± 1.50^c^	−2.54 ± 0.53^ab^
	24 h	5.35 ± 1.15^d,B^	2.02 ± 0.58^c,B^	1.46 ± 1.27^bc,A^	−0.45 ± 1.61^abc,A^	−1.75 ± 0.89^ab^	1.21 ± 0.65^bc^	−1.25 ± 0.69^abc^	−0.65 ± 0.94^abc^	−2.91 ± 1.46^a^	−2.60 ± 0.60^a^
	48 h	13.06 ± 2.55^d,C^	2.58 ± 0.57^b,B^	8.55 ± 1.10^c,B^	5.92 ± 1.52^bc,B^	−4.25 ± 0.98^a^	2.62 ± 1.23^b^	−3.30 ± 1.29^a^	−1.64 ± 1.16^a^	−1.90 ± 1.56^a^	−3.59 ± 0.68^a^
Cooking loss (%)	2 h	33.75 ± 1.13	29.87 ± 2.36^A^	35.56 ± 1.05	34.38 ± 1.39	32.97 ± 2.40^A^	33.82 ± 3.38	35.44 ± 2.85	34.19 ± 2.69	30.92 ± 2.40^A^	30.98 ± 2.27
	24 h	31.20 ± 2.79^ab^	36.47 ± 1.52^bc,B^	37.67 ± 1.26^c^	39.14 ± 1.85^c^	34.21 ± 1.69^bc,A^	33.74 ± 1.54^abc^	36.99 ± 1.73^c^	36.85 ± 1.22^bc^	35.12 ± 1.79^bc,AB^	28.71 ± 1.97^a^
	48 h	30.62 ± 1.59^a^	34.48 ± 1.44^b,AB^	37.36 ± 1.82^bc^	37.51 ± 1.56^bc^	39.36 ± 0.82^c,B^	37.95 ± 0.78^bc^	39.15 ± 0.97^c^	39.64 ± 0.84^c^	39.38 ± 1.28^c,B^	29.33 ± 0.94^a^
TBARS (mg MDA/kg)	2 h	0.13 ± 0.04	0.18 ± 0.05	0.22 ± 0.06	0.20 ± 0.06	0.10 ± 0.02	0.10 ± 0.01	0.14 ± 0.02	0.12 ± 0.01	0.22 ± 0.07	0.16 ± 0.01^A^
	24 h	0.32 ± 0.13	0.37 ± 0.13	0.38 ± 0.13	0.34 ± 0.12	0.10 ± 0.01	0.12 ± 0.02	0.15 ± 0.03	0.15 ± 0.02	0.51 ± 0.19	0.23 ± 0.02^AB^
	48 h	0.20 ± 0.04^bc^	0.13 ± 0.01^abc^	0.13 ± 0.02^abc^	0.09 ± 0.02^a^	0.10 ± 0.02^a^	0.11 ± 0.01^ab^	0.12 ± 0.01^ab^	0.14 ± 0.02^abc^	0.21 ± 0.06^c^	0.30 ± 0.04^d,B^

*Note:* a–e, mean values with different lower case letters differ significantly among samples (*p* < 0.05). A–C, mean values with different upper case letters differ significantly among marination times (*p* < 0.05).

Cooking loss values of the samples were significantly influenced by the utilization of UGJ or DUGP during the marinating process and the marination holding time. Furthermore, cooking losses of samples demonstrated compatibility with the pH values. The pH values of all the samples marinated with UGJ increased until the 24th hour, after which they decreased at the 48th hour, except for the BML1 sample (*p* < 0.05). In accordance with this result, although statistically insignificant, the cooking loss values of all samples except BML1 increased until the 24th hour and then decreased until the 48th hour. It was observed that the most suitable marinating time in terms of cooking loss value for the BML1 sample was 48 h. A different situation was observed in the samples marinated with DUGP. The pH values of all samples increased significantly during the marination process (*p* < 0.05). In accordance with the pH values, the cooking loss values of all samples marinated with DUGP increased during marination, and the highest cooking loss values were observed at 48th hour. This increase was statistically significant in the BML5 sample (*p* < 0.05). The decrease in pH values of the samples, caused by the UGJ and DUGP, resulted in the samples retaining more liquid in their structures, which led to a decrease in cooking losses. The pH of the samples shifted away from the isoelectric point as a result of the acidic marinade, leading to an increase in marinade absorption value and the retention of more liquid within the meat structure (Yusop et al. 2010).

The TBARS test is employed to quantify malondialdehyde, which serves as the primary indicator of lipid oxidation that causes the final product to lose its nutritional and sensorial properties (Alarcon et al. 2021). In the present study, the TBARS values of the samples exhibited a range of 0.09–0.51 mg MA/kg sample (Table 4). Although no notable distinction was observed in TBARS values between the samples at the 2 h and 24 h marination intervals (*p* > 0.05), a marked divergence emerged at the 48 h of holding time. This was attributed to the efficacy of unripe grape products in impeding the oxidation process. Given that the rate of oxidation is dependent on the duration of processing or storage in meat and meat products, the discrepancies between samples become more pronounced at the 48 h mark, which represents the longest processing time. The TBARS values of all marinated samples were significantly lower than those of the control sample bc2 (*p* < 0.05) and the TBARS values of BML4, BML5, BML6, and BML7 were found to be significantly lower than those of the control sample bc1 at the 48 h of holding time (*p* < 0.05). The antioxidant activity of unripe grapes is attributed to their high concentrations of phenolic and polyphenolic compounds, including gallic acid, caffeic acid, catechin, and quercetin glycoside (Nikfardjam 2008; Turkmen et al. 2017; Ozturk and Sengun 2019). The antioxidant capacity can be explained by the fact that the phenolic compounds are able to deactivate and stabilize free radicals by incorporation into their aromatic ring and to absorb UV light (Brewer 2011; Christaki et al. 2012; Maqsood et al. 2014; Mantzourani et al. 2023). Some fiber compounds in grape pomace make chemical bonds with phenolic substances and, thus, create antioxidant dietary fibers, giving the pomace stronger radical scavenging potential (Antonic et al. 2020).

### 3.4 Effect of Marination on Color Properties

**FIGURE 1:**
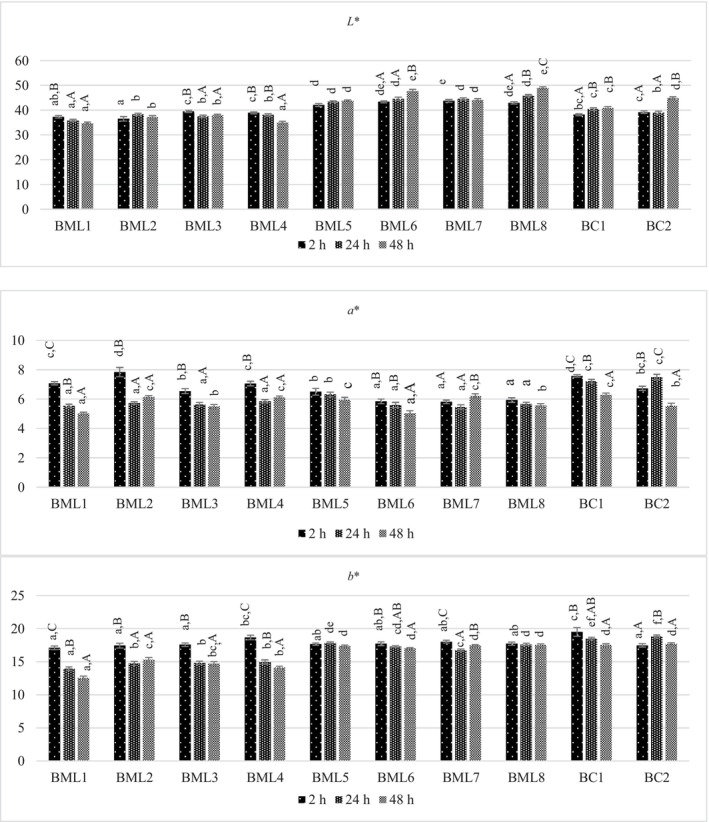
Influence of marinade formulations and marination holding times on color characteristics of beef samples. *a–f; Mean values with different lower case differ significantly among samples (*p* < 0.05). A–C; Mean values with different upper case differ significantly during marination times (*p* < 0.05).

The incorporation of unripe grape products resulted in a notable impact on the color parameters of the samples (Figure 1). The marinated BML6 and BML8 samples, prepared with thyme and salt in conjunction with DUGP for a period of 48 h, exhibited elevated L* values in comparison to the control samples (*p* < 0.05). This observation was also evident in the marinated samples that were subjected to the same marinating formulation for 2 and 24 h, respectively. This may be attributed to the antioxidant effect of DUGP and thyme, which may have prevented the darkening of meat samples caused by oxidation during the marination process. In accordance with this result, Lee et al. (2017) determined that the L* values of the pork loins marinated with grape pomace in different ratios were significantly higher in all treatments compared with those of the control group.

The L* values of the samples marinated with UGJ were found to be lower than those of the samples marinated with DUGP (*p* < 0.05). The darkening effect of the marinades containing UGJ on the samples was attributed to both the characteristic greenish color and the lower ratio of water used in place of UGJ in the marinade formulations. Conversely, in the formulations of marinades with DUGP, a higher amount of water resulted in a brighter color.

The L* values of the samples marinated for 48 h with UGJ were found to be significantly lower in comparison to the 2 h holding time (*p <* 0.05). Therefore, the unique greenish color of UGJ exerted a greater influence on the color of the meat with higher marinade absorption during longer marination time. Furthermore, the L* values of the BML6 and BML8 samples marinated with a marinade containing thyme and DUGP for 48 h exhibited significantly higher values than those of the samples marinated for 2 h. This is attributed to the antioxidant effect of thyme and DUGP.

The a* values of all marinated samples were found to be significantly lower than those of the control samples for a holding time of 24 h (*p* < 0.05). Upon extending the marination period to 48 h, the a* values of the samples approached those of the control samples (*p >* 0.05). This phenomenon may be attributed to the dissolution of myoglobin, the pigment responsible for the red coloration of meat, in water over an extended period of time. This process results in a reduction in the perceived redness of the meat. Furthermore, the elevated acidity value of UGJ may potentially lead to an increased denaturation of myoglobin protein, contingent on the marination duration. In a study conducted by Tkacz et al. (2021), marination of beef with pepper and garlic resulted in a decrease in L* and a* values of the samples. In contrast, in another study performed by Sengun et al. (2021), the a* values of beef samples marinated with rosehip and grape vinegars were lower than those of samples marinated with blackberry and pomegranate vinegars.

The b* values of the samples treated with UGJ for 24 and 48 h were found to be significantly lower than those of the samples treated with DUGP (*p* < 0.05). The addition of greenish UGJ to the marinade in place of water resulted in a reduction in the yellowish tone (b* value) of beef samples at longer marination times. Similarly, a study conducted by Alarcon et al. (2021) demonstrated that the treatment of a cooked pork model system with grape stem and vine‐shoot extracts resulted in lower b* values of the samples.

### 3.5 Effect of Marination on Texture Profile

**FIGURE 2:**
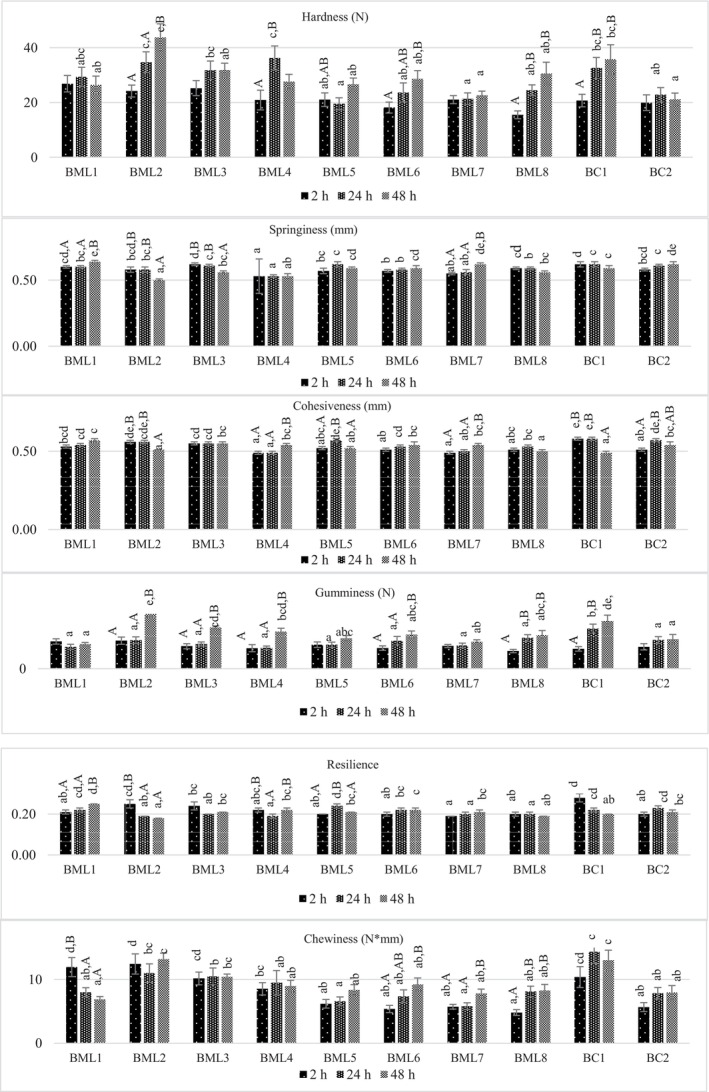
Influence of marinade formulations and marination holding times on texture profile of beef samples. *a–f; Mean values with lower case differ significantly among samples (*p* < 0.05). A–C; Mean values with different upper case differ significantly during marination *p* < 0.05).

It was determined that the hardness values of the BML5 and BML7 samples were lower than those of the bc1 control sample after 24 h of marinating (*p <* 0.05) (Figure 2). One hypothesis for the tenderizing effect of acidic marinades suggests that the swelling of muscle fibers and connective tissue leads to a dilution of load‐resisting material, resulting in a maximum level of tenderness and swelling under identical conditions (Offer and Knight 1988). A secondary hypothesis is that the optimal pH for the activity of cathepsins is in the range 3.5–5.0. It can thus be concluded that the lowering of meat pH in an acid marinade may possibly enhance proteolytic attack by these enzymes (Burke and Monahan 2003).

When the marination time increased to 48 h, a decrease in the hardness value of the BML4 was detected (*p <* 0.05). This desired decrease in the hardness of the BML4 was not observed in other sample groups at 48 h. Therefore, in terms of hardness value, it appears that the marinating time of all samples except BML4 should be 24 h. Similarly, Karatepe et al. (2023) detected that the most effective treatment was at 24 h with 100% hawthorn vinegar in terms of reduced beef hardness values in comparison to untreated beef. The application of low pH marinades to meat can result in the denaturation of the meat surface, thereby preventing the progression of marinade penetration over an extended marinating holding period. In such instances, marinades are unable to permeate the deeper layers of muscle tissue (Tarantino 2006; Yusop et al. 2010). Therefore, when the marination time is prolonged, the desired textural effects may not be seen. In line with this, Kaewthong et al. (2021) detected that the lowest hardness and chewiness of marinated dairy‐goat meat was obtained at a marinating time of 60 min with both pineapple and ginger juices according to 30 and 90 min marinating times.

It was detected that the hardness value of the BML2 that marinated with UGJ for 24 and 48 h was significantly higher than all the DUGP treated samples and the bc2 control sample (*p <* 0.05). A low pH value (e.g., 4.1) in marinated meat can result in protein denaturation, which in turn increases meat hardness (Yusop et al. 2010). It can thus be concluded that the meat marinated with UGJ exhibited higher hardness values than the other samples, likely due to the low pH. Similarly, in a study by Beltrán‐Cotta et al. (2023), higher hardness values were reported for samples marinated in yellow mombin ( *Spondias mombin* L.) for 20 h compared to the control sample on the 7th day of storage (*p <* 0.05).

As the marination holding time increased, the chewiness value of the BML1 decreased, while those of the BML6, BML7, and BML8 increased (*p* < 0.05). The gumminess values of the samples increased with the duration of marination. Consequently, the gumminess values of the BML2, BML3, BML4, BML6, BML8, and bc1 samples, which were marinated for 48 h, were significantly higher than those of the samples marinated for 2 h (*p* < 0.05).

The effects of different marinade additives and marination time cannot be clearly seen in some of the textural characteristics of the samples. One reason for this situation is thought to be the changing interaction of different parts of the beef, such as fat and connective tissue, with the marinade during the marinating process in stagnant liquid. The other reason was attributed to the combination effects of unripe grape products with other additives during different marination times, resulting in fluctuations in some of the texture characteristic results.

### 3.6 Effect of Marination on the Microbiological Quality of Meat Samples

**FIGURE 3:**
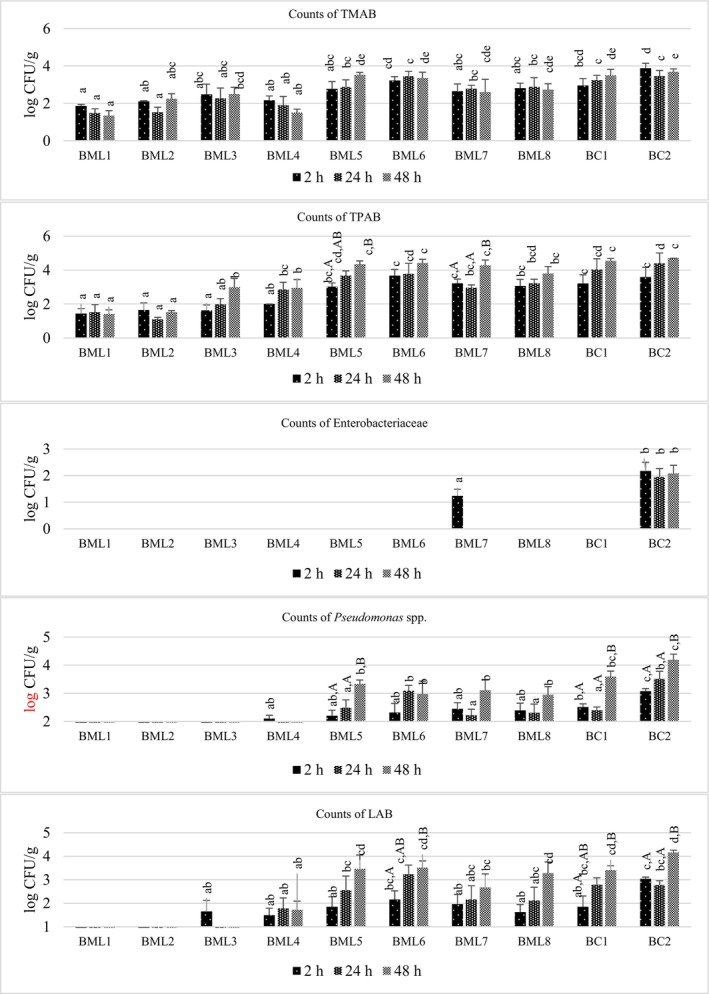
Influence of marinade formulations and marination holding times on microbiological properties of beef samples. *a–e; Mean values with different lower case differ significantly among samples (*p* < 0.05). A, B; Mean values with different upper case differ significantly during marination times (*p* < 0.05).

TMAB counts are commonly used as a standard to determine whether meat is microbiologically contaminated (Cohen et al. 2007). After 2, 24, and 48 h at 4°C, TMAB counts of untreated meat samples (bc2) in the study were 3.88, 3.46, and 3.69 log colony forming unit (CFU)/g, respectively (Figure 3). These are the initial microbial counts of untreated meat samples; they are used as a control to compare how marination affects the samples. At 2, 24, and 48 h of marination, ML1 effectively reduced the TMAB counts of the meat samples; however, at 2 and 24 h of marination, ML2 was more successful than ML1, with reductions of 1.77 and 1.94 log CFU/g, respectively (*p <* 0.05) (Figure 1). However, the inhibitory effect of MLs against TMAB decreased in parallel with the decrease of UGJ concentration in the formulation of MLs. Among the MLs prepared with DUGP after 2, 24, and 48 h of marination, ML7 was the most effective formulation, reducing the TMAB count by 1.22, 0.68, and 1.09 log CFU/g, respectively (Figure 1). These results clearly demonstrated that the inhibitory effects of MLs prepared with UGJ on the TMAB counts of the samples were higher than those of MLs prepared with DUGP for all time applications (*p <* 0.05). Furthermore, the effects of all MLs on TMAB of the samples were not significantly different (*p >* 0.05) for all application times (Figure 3).

Sengun et al. (2019) investigated the effects of MLs prepared with koruk juice on the quality of poultry meat. They marinated the samples at 4°C for 1, 2, and 18 h and found that TMAB counts were below the detection limits for all samples marinated with 100% koruk juice. The differences can be attributed to the type of meat used in the study, microbial load, and the concentration of koruk juice. Another study investigated the total viable count of pork loin marinated in grape (*V. labruscana* Bailey) pomace (20% and 40%) and powder (0.5%, 1% and 2%) at 4°C for 72 h and vacuum‐packed at 4°C for 10 days. They concluded that, similar to our result, the total viable count decreased as the concentration of grape pomace and grape pomace powder increased (Lee et al. 2017). These findings indicate that grape products exert a beneficial effect on the microbial quality of different types of meat.

The TPAB count is considered an important indicator of meat preservation quality and is used to predict meat shelf life and spoilage at refrigerator temperature (McEvoy et al. 2000). However, it is crucial for assessing the refrigerated shelf life of meat. In this study, the effect of MLs on TPAB found in aerobically stored meat in the refrigerator was determined. The TPAB counts of the samples were reduced by MLs prepared with UGJ in the range of 1.58–2.15, 1.54–3.28, and 1.71–3.29 log CFU/g after 2, 24, and 48 h, respectively (Figure 1). The highest reduction in TPAB count was achieved by marinating with ML1 for 2 and 48 h treatment and ML2 for 24 h treatment (*p >* 0.05). However, among the MLs prepared with DUGP, ML5 for 2 h, ML7 for 24 h, and ML8 for 48 h were the most effective treatments by reducing the TPAB count by 0.56, 1.43, and 0.90 log CFU/g, respectively (*p <* 0.05). For time‐related applications, there was a significant difference between the effects of ML5 and ML7 among all MLs on TPAB counts in meat samples (*p >* 0.05) (Figure 3).

The presence of Enterobacteriaceae at high levels (> 3 log CFU/g) in meat indicates inadequate sanitary conditions (Arcanjo et al. 2019). In the study, the Enterobacteriaceae count of bc2 was 2.17 log CFU/g after 2 h. However, the Enterobacteriaceae counts found in meat samples were completely inactivated by all MLs except ML7 for the 2 h application. For the 24 and 48 h applications, the count of Enterobacteriaceae found in BML7 was also reduced below the detection limit (*p <* 0.05) (Figure 3).

Ortega‐Heras et al. (2020) aimed to prepare low‐salt marinated chicken breasts by adding a seasoning obtained from red grape skins (0.5% and 2%). The marinated breasts were stored under refrigeration, and they found that the presence of the seasoning in the brines reduced the growth of Enterobacteriaceae. An increase in the ratio used may result in a decline in Enterobacteriaceae count; however, this may also have an unfavorable impact on sensory acceptability.

*Pseudomonas* spp. are associated with the spoilage of raw meat under aerobic conditions due to their high growth rate (Borch et al. 1996). In this study, the count of *Pseudomonas* spp. found in bc2 (3.07, 3.51, and 4.19 log CFU/g after 2, 24, and 48 h, respectively) was completely inactivated by ML1, ML2, and ML3 for all time applications (*p <* 0.05) (Figure 1). The most successful formulation among the other MLs was ML4, which reduced the *Pseudomonas* spp. counts of BML4 to 2.10 log CFU/g after 2 h and below the detection limit for the next 24 and 48 h at 4°C (*p <* 0.05). Similarly, among the MLs prepared with DUGP, the highest reductions were achieved by ML5, ML7, and ML8 for 2, 24, and 48 h marination, respectively (Figure 1). Furthermore, there was no significant difference (*p >* 0.05) between the time applications of ML1, ML2, ML3, ML4, ML6, ML7, and ML8 on the *Pseudomonas* spp. counts of beef samples (Figure 3). Although LAB is used to maintain and achieve a desired flavor in fermented meat products, it is undesirable and leads to spoilage in raw meat (Vasilijević et al. 2019). The counts of LAB found in bc2 were 3.03, 2.77, and 4.16 log CFU/g after 2, 24, and 48 h, respectively, while these levels were reduced below detection limits by ML1 and ML2 containing high levels of UGJ for all time applications (*p <* 0.05) (Figure 3). When meat samples were marinated with ML3, the counts were reduced to 1.65 log CFU/g after 2 h and below the detection limit at 4°C for 24 and 48 h. The count of LAB found in BML4 was 1.50, 1.78, and 1.72 log CFU/g after 2, 24, and 48 h at 4°C, respectively. However, the counts of LAB found in BML5, BML6, BML7, and BML8 were 1.63 to 2.16, 2.16 to 3.23, and 2.68 to 3.51 log CFU/g after 2, 24, and 48 h at 4°C, respectively (*p <* 0.05) (Figure 3).

In a study, wine‐based marinades containing ethanolic extract from pomegranate ( *Punica granatum* L.), alone or in combination with two essential oils (Thyme and Oregano), were used for pork fillets marination at 4°C for 1 h. Following the marination, the samples were stored at 4°C for 7 days. They stated that LAB counts increased during storage. These findings are similar to the LAB results in the samples marinated with DUGP in our study (Mantzourani et al. 2023). These results showed that LABs found in meat could be resistant to the grape‐based products. Moreover, these products can support the growth of LAB, suggesting a potential prebiotic effect (Pistol et al. 2019). When compared to UGJ, DUGP appears to have a more pronounced effect on LAB proliferation, possibly due to the unique composition of the grape‐based products.

In the literature, there is no study investigating the effects of unripe grape‐based MLs on the microbiological quality of beef meat. However, in our previous study, the inactivation effects of MLs prepared with UGJ juice and DUGP against *Escherichia coli* O157:H7, *Salmonella Typhimurium* , and *Listeria monocytogenes* inoculated on beef meat were determined, and the counts of *Escherichia coli* O157:H7, *S. Typhimurium* , and *L. monocytogenes* on the samples were reduced by 0.11–2.65, 0.26–3.37, and 0.02–2.78 log CFU/g, respectively (Ozturk and Sengun 2019). This result demonstrates that koruk products have the potential to be utilized in meat marinades, thereby ensuring meat safety. In another study, the effects of MLs prepared with UGJ on *S. typhimurium* inoculated on poultry meat and on some quality attributes of poultry meat were investigated. *S. Typhimurium* and TMAB counts of the samples were reduced by MLs in the range of 0.11–3.38 and 1.04–2.56 log CFU/g, respectively (Sengun et al. 2019). These studies showed that UGJ and DUGP have an important potential to increase the safety of meat, and their efficacy could vary depending on the type of meat, concentration of UGJ and DUGP, and exposure time used.

In the literature, research has shown how the marinating technique affects the microbiological quality of meat. For example, in a study, the effect of ML prepared with three different wines (Carbernet (CAB), Tempranillo (TEM) and Isabel (ISA), 300 mL dealcoholized wine/kg meat) on total viable bacteria (TVB), Enterobacteriaceae, and LAB found in meat was determined during 7 days of storage at 4°C. The highest inhibitory effect of the marination process on the TVB count was observed on the 5th day; a significant reduction of up to 1.4 log CFU/g was detected in the samples compared to the control (treated with distilled water). The reduction of Enterobacteriaceae levels (approximately 3 log CFU/g) was observed in all treatments after 3 days of storage, while LAB counts showed an increase during the storage time (Arcanjo et al. 2019). Similarly, in this study, the count of Enterobacteriaceae found in meat samples was completely inactivated by ML prepared with UGJ and DUGP for 24 and 48 h applications. In contrast, the count of LAB found in meat samples marinated with DUGP showed a significant increase during the application time (Figure 3). This finding suggests that DUGP may have favored the growth of LAB.

The results of our study also showed that ML prepared with UGJ is more effective on the counts of TMAB, TPAB, *Pseudomonas* spp., Enterobacteriaceae, and LAB found in meat samples than ML prepared with DUGP (Figure 3). All these studies showed that the efficacy of marination is directly dependent on the marination formulation (content, concentration, ingredients used), the type of meat, temperature, and application times used.

### 3.7 Effect of Marination on Sensory Properties

**FIGURE 4:**
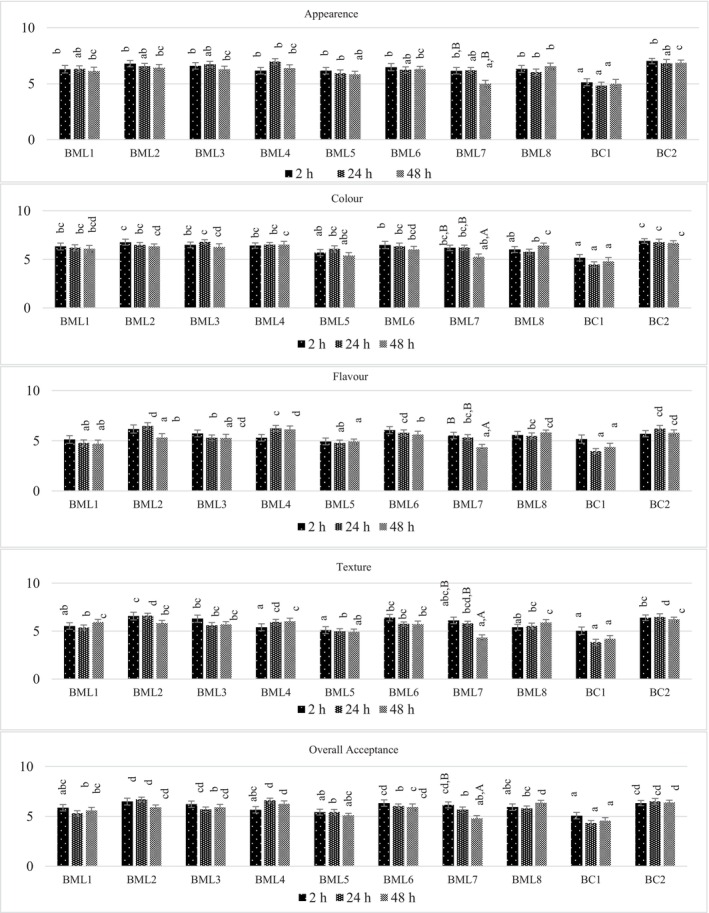
Influence of marinade formulations and marination holding times on sensory evaluation scores of beef samples. *a–e; Mean values with different lower case differ significantly among samples (*p* < 0.05). A, B; Mean values with different upper case differ significantly during marination times (*p* < 0.05).

Color, texture, flavor, and overall acceptance of beef samples improved with the use of UGJ and DUGP in marinating. All marinated sample groups had significantly higher color, texture, and overall acceptance scores compared to control bc1 when marinated for 24 h (*p* < 0.05) (Figure 4). As a result of 48 h marination, all UGJ marinated samples, as well as BML6 and BML8, had significantly higher appearance, color, and texture scores compared to bc1 (*p* < 0.05).

BML5 and BML7, which do not contain thyme in the marinade formulations, had similar color and appearance scores to bc1 (*p* > 0.05). This result shows that the unripe grape products and thyme in the marinades were more effective in slowing down the oxidation reaction that affects the color. The inhibition of myoglobin oxidation resulted in the appearance of the meat being better preserved during the marinating process. Although the appearance and color scores of all samples decreased after 24 h of marination, only BML7 showed a statistically significant decrease (*p* < 0.05).

Flavor scores of samples treated with UGJ were found to be higher than the control bc1 at all marinating times. Similar to the appearance and color scores, although the flavor scores of all samples decreased after 24 h of marination, only the decrease in the scores of BML7 was statistically significant (*p <* 0.05). It was concluded that the 24 h marination process gave more positive sensory evaluation results compared to the 2 h, but 24 h should not be exceeded.

Considering all the sensory characteristics of the samples, the samples treated with marinades containing UGJ and DUGP with thyme improved the characteristics of the beef samples. Following a 24 h marinating period, the overall acceptance scores of BML2 (6.7) and BML4 (6.6) were higher than all other marinated samples and the control sample bc1 (4.33), with the exception of BML6 (6.03) (*p <* 0.05). The content and flavor of unripe grape products were thought to be effective in improving the beef flavor. Unripe grapes contain little simple sugar but are rich in organic acids, phenolic flavonoids, non‐flavonoids, condensed tannins, stilbenes, and glutathione (Adams 2006). In addition to the unique flavor of the unripe grape products used in marination improving the flavor of the meat, inhibition of oxidation and the textural properties that change within the framework of the acidic marination application principle also positively affected the flavor. Thyme also had a positive effect on the sensory properties of the marinated beef samples due to its antioxidant properties, slowing down oxidation and enabling the meat to better preserve its characteristics and its unique pleasant flavor. The antioxidant activity of thyme is attributed to thymol and carvacrol, present in thyme essence, as well as the flavonoids and other polyphenols (Hailemariam and Emire 2013). In accordance with these results, Mantzourani et al. (2023) determined that the flavor of pork loin samples marinated with pomegranate extract and red wine improved positively compared to samples marinated without the addition of pomegranate extract. Similarly, Kaewthong et al. (2021) found that the samples marinated with pineapple juice for 60 min and then with a barbecue sauce containing 3% sodium bicarbonate for 60 min had less cooking loss and hardness, and scored higher for all sensory attributes than the non‐juice‐marinated meat with sodium bicarbonate and the ginger‐marinated meat. There are studies that reported that sensory properties were improved with the use of different herbal additives in the acidic marinating process of beef, such as citrus juice (Burke and Monahan 2003), thymol and carvacrol added vinegar‐based marinade (Karam et al. 2020), blackberry, pomegranate, rosehip, and grape vinegar (Sengun et al. 2021).

## 4 Conclusion

The use of UGJ and DUGP improved the physical, chemical, sensory, and microbiological characteristics of beef depending on the application rate and marination time. Thus, one of the objectives of this study, which was to improve the microbiological and oxidative quality of beef through natural marinade additives while developing its sensory properties, was achieved through the application of UGJ and DUGP. The second objective of this study, to determine the most appropriate marinating time for each marinade formulation, was also achieved for all product characteristics studied. Using the highest amount of UGJ (50%) in the marinade formulations resulted in the highest acidity, moisture content, and marinade absorption values in BML1 at the end of the 24 and 48 h marination times. All unripe grape products were found to be effective in slowing oxidation in beef samples during the 48 h of holding time. Although the use of UGJ in the marination formulations caused a decrease in the L* values of the samples, as a result of the sensory evaluation, it was determined that the color scores of all samples using UGJ were higher than those of the control bc1. Marinating the samples with the formulation containing 50% UGJ (BML1) for 48 h was more effective in reducing TMAB count than marinating with DUGP. Furthermore, marinating with formulations containing 50% UGJ resulted in the reduction of *Pseudomonas* and LAB count to below the detection limit. Furthermore, it has been demonstrated that MLs containing UGJ are more effective than those containing DUGP in terms of providing microbial inactivation or inhibition. All the sensory characteristics (color, texture, flavor and overall acceptance) of beef were improved with marinades containing UGJ and DUGP with thyme. It could be suggested that a 24 h holding time for beef marinades with unripe grape products should not be exceeded in terms of sensory characteristics. As a result of this study, it was concluded that UGJ and DUGP can be used as a natural and healthy marinating component to obtain beef with improved quality in terms of physicochemical, microbiological, and sensorial. The results obtained in this study can be transferred to the industry, and unripe grape products can be used as natural and healthy additives in beef marinades. Thus, a new evaluation area will arise in the food industry for unripe grapes, which possess antimicrobial and antioxidant properties and have beneficial effects on health. Future studies could be directed towards investigating the potential of using unripe grape products as additives in the formulation of various foodstuffs in order to provide new natural additive alternatives to the food industry, thus serving the issues of waste utilization and proper use of resources and to maintain the nutritional value and improved health effects of these products with novel techniques applied in the food industry.

## Author Contributions

**Gulen Yildiz Turp:** conceptualization (equal), investigation (equal), methodology (equal), project administration (equal), supervision (equal), validation (equal), writing – original draft (equal), writing – review and editing (equal). **Ilkin Yucel Sengun:** conceptualization (equal), investigation (equal), methodology (equal), project administration (equal), supervision (equal), validation (equal), writing – original draft (equal), writing – review and editing (equal). **Gulden Kilic:** investigation (equal), methodology (equal), validation (equal), writing – original draft (equal). **Şeyma Nur Şeker:** investigation (equal), methodology (equal), validation (equal), writing – original draft (equal). **Berna Ozturk:** investigation (equal), methodology (equal), validation (equal), writing – original draft (equal). **Aysegul Kirmizigul Peker:** investigation (equal), methodology (equal), validation (equal), writing – original draft (equal), writing – review and editing (equal).

## Ethics Statement

No ethical document was required for the sensory analysis at the time this project was carried out, as confirmed by the Science and Engineering Sciences Scientific Research and Publication Ethics Committee, Ege University.

## Consent

All authors have consented for the publication of this study.

## Conflicts of Interest

The authors declare no conflicts of interest.

## Data Availability

Data supporting the findings of this study are available upon request from the corresponding author.
